# Type I Interferons and Interferon Regulatory Factors Regulate TNF-Related Apoptosis-Inducing Ligand (TRAIL) in HIV-1-Infected Macrophages

**DOI:** 10.1371/journal.pone.0005397

**Published:** 2009-04-30

**Authors:** Yunlong Huang, Angelique Walstrom, Luwen Zhang, Yong Zhao, Min Cui, Ling Ye, Jialin C. Zheng

**Affiliations:** 1 Laboratory of Neurotoxicology, Department of Pharmacology and Experimental Neuroscience, University of Nebraska Medical Center, Omaha, Nebraska, United States of America; 2 Department of Pathology and Microbiology, University of Nebraska Medical Center, Omaha, Nebraska, United States of America; 3 China-U. S. Joint Research Center for Life Sciences, Beijing, China; 4 Nebraska Center for Virology, School of Biological Sciences, University of Nebraska, Lincoln, Nebraska, United States of America; 5 Transplantation Biology Research Division, State Key Laboratory of Biomembrane and Membrane Biotechnology, Institute of Zoology, Chinese Academy of Sciences, Beijing, China; New York University School of Medicine, United States of America

## Abstract

TNF-related apoptosis-inducing ligand (TRAIL) is a member of the TNF family that participates in HIV-1 pathogenesis through the depletion of CD4+ T cells. TRAIL is expressed on the cell membrane of peripheral immune cells and can be cleaved into a soluble, secreted form. The regulation of TRAIL in macrophages during HIV-1 infection is not completely understood. In this study, we investigated the mechanism(s) of TRAIL expression in HIV-1-infected macrophages, an important cell type in HIV-1 pathogenesis. A human monocyte-derived macrophage (MDM) culture system was infected with macrophage-tropic HIV-1_ADA_, HIV-1_JR-FL_, or HIV-1_BAL_ strains. TRAIL, predominantly the membrane-bound form, increased following HIV-1 infection. We found that HIV-1 infection also induced interferon regulatory factor (IRF)-1, IRF-7 gene expression and signal transducers and activators of transcription 1 (STAT1) activation. Small interfering RNA knockdown of IRF-1 or IRF-7, but not IRF-3, reduced STAT1 activation and TRAIL expression. Furthermore, the upregulation of IRF-1, IRF-7, TRAIL, and the activation of STAT1 by HIV-1 infection was reduced by the treatment of type I interferon (IFN)-neutralizing antibodies. In addition, inhibition of STAT1 by fludarabine abolished IRF-1, IRF-7, and TRAIL upregulation. We conclude that IRF-1, IRF-7, type I IFNs, and STAT1 form a signaling feedback loop that is critical in regulating TRAIL expression in HIV-1-infected macrophages.

## Introduction

TNF-related apoptosis-inducing ligand (TRAIL) is a member of the TNF superfamily and an important immune regulatory factor capable of inducing apoptosis [1]–[3]. TRAIL is expressed on the cell membrane of CD4+ T lymphocytes, natural killer cells, and mononuclear phagocytes (monocytes and macrophages) and can be cleaved into a soluble, secreted form [4]. The plasma levels of TRAIL are increased in HIV-1-infected patients compared to uninfected individuals, and patients receiving anti-retroviral therapy show decreased plasma TRAIL levels that correlate with reduced viral load [5]. Increased TRAIL expression is an important contributor to HIV-1-mediated apoptosis in bystander CD4+ T cells [6]–[9]. Furthermore, recombinant human TRAIL has been found to induce apoptosis in HIV-1-infected macrophages and cultured neurons as we have previously reported [10], [11]. Although the apoptotic signaling events of TRAIL have been studied extensively, including our recent work [10]–[13], the upstream molecular stimuli, particularly those that are responsible for HIV-1-mediated TRAIL upregulation, remain unclear.

Macrophage (M)-tropic HIV strains preferentially infect mononuclear phagocytes, a cell type critical to HIV-1 replication in the disease [14], [15]. Infected mononuclear phagocytes disseminate virus to lymph nodes where CD4+ T lymphocytes become infected and to tissues, including the lung and central nervous system, where they serve as viral reservoirs [16]–[19]. TRAIL expression is induced by interferon (IFN)-α, -β, and -γ in monocytes [20], by IFN-α and -β but not -γ, in Jurkat cells [21], and by IFN-β in CD4+ T cells [22]. However, limited information on how HIV-1 regulates TRAIL in mononuclear phagocytes has been reported to date.

Type I IFNs IFN-α and -β are primarily induced by plasmacytoid dendritic cells (pDCs) and in a lower amount by monocytes and macrophages following viral infection [23], [24]. All type I IFNs interact with the IFN-α receptor (IFNAR), which appears to couple to a uniform signal transduction cascade (for review, see [25]). IFN-α/β binding triggers receptor dimerization and activation, leading to phosphorylation of a tyrosine residue on IFNAR. This phosphorylation stimulates the JAK/STAT pathway leading to the formation of signal transducers and activators of transcription 1 (STAT1) homodimers as well as heterodimers with STAT3 [26]. Activated STAT dimers translocate to the nucleus and bind to interferon stimulated response elements of the promoters for IFN-stimulated genes(for review, see [27]), including TRAIL [28].

IFN regulatory factors (IRFs) are a family of transcription factors that regulate the antiviral response. IRFs are closely related to type I IFNs and consist of nine mammalian proteins characterized by an amino-terminal DNA-binding domain [29]. Gene knockout of IRF-1, IRF-3, or IRF-7 results in high susceptibility to infectious agents [30]–[32]. IRF-1 and IRF-7 were identified by their ability to induce the transcription of type I IFN and IFN-inducible genes, and both are induced by HIV-1 [33]–[36]. IRF-1, IRF-3, and IRF-7 are constitutively expressed in most cell types, whereas the expression of IRF-1 and IRF-7 are more inducible following exposure of cells to IFNs [37]. In addition, IRF-1, IRF-3, and IRF-7 have been linked to TRAIL transcription [38]–[41]. However, the exact role of IRFs in the promotion of TRAIL expression, especially in mononuclear phagocytes, remains unclear.

Using a monocyte-derived macrophage (MDM) model, we investigated how TRAIL expression is upregulated in macrophages during HIV-1 infection. Our results demonstrated that upregulation of TRAIL expression in HIV-1-infected MDM was predominantly membrane-associated. HIV-1 infection induced IRF-1 and IRF-7 gene expression and STAT1 phosphorylation in macrophages. Small interfering RNA (siRNA) knockdown of IRF-1 or IRF-7 but not IRF-3 reduced STAT1 activation and TRAIL expression. Treatment with various cytokines identified IFNs as the critical factors stimulating TRAIL expression. The upregulation of IRF-1, IRF-7, and TRAIL, and the activation of STAT1 by HIV-1 infection was reduced by the treatment of type I interferon neutralizing antibodies. In addition, inhibition of STAT1 by fludarabine abolished IRF-1, IRF-7, and TRAIL upregulation. These data suggest that IRF-1, IRF-7, Type I IFNs, and STAT1 form a signaling feedback loop and cooperatively regulate TRAIL expression in macrophages during HIV-1 infection. Understanding the signaling events in HIV-1-infected macrophages may lead to the development of new therapies to alleviate macrophage-mediated HIV-1 pathogenesis by reducing the expression of death ligand TRAIL.

## Materials and Methods

### Reagents

Recombinant proteins, neutralizing antibodies, and chemicals were obtained as follows: IL-1β, TNF-α, IFN-γ, IFN-β neutralizing antibody, and Mouse IgG1 (R&D Systems, Minneapolis, MN); IFN-α, IFN-β, and IFN-α neutralizing antibody (PBL Interferon Source, Piscataway, NJ); Aidovudine (AZT, HIV-1 reverse transcriptase inhibitor), fludarabine, and lipopolysaccharide (LPS) (Sigma-Aldrich, St. Louis, MO).

### Monocyte cell culture and HIV-1 infection

Human monocytes were isolated from peripheral blood mononuclear cells of HIV-1, -2, and hepatitis B seronegative donors after leukopheresis and counter current centrifugal elutriation [42]. Monocytes were cultured as adherent monolayers at a density of 1.1×10^6^ cells/well in 24-well plates and cultivated in Dulbecco's modified Eagles medium (DMEM, GIBCO Invitrogen Corp, Carlsbad, CA) with 10% heat-inactivated pooled human serum (Cambrex Bio Science, Walkersville, MD), 50 µg/ml gentamicin, 10 µg/ml ciprofloxacin (Sigma-Aldrich, St. Louis, IL), and 1000 U/ml highly purified recombinant human macrophage colony stimulating factor (M-CSF) (a generous gift from the Wyeth Institute, Cambridge, MA).

Seven days after plating, MDM were infected with HIV-1 strains ADA, BAL, or JR-FL at a multiplicity of infection (MOI) of 0.1. On the second day after infection, media were removed and substituted with MDM culture media (DMEM with 10% heat-inactivated pooled human serum, 50 µg/ml gentamicin, and 10 µg/ml ciprofloxacin) [42]. Stock virus was screened for mycoplasma and endotoxin using hybridization and Limulus amebocyte lysate assays, respectively. Five days after infection, cells were changed to 0.5 ml/well fresh medium for 24 hours. Culture supernatants were obtained and subsequently stored at −80°C until assayed. Filtration of supernatants was performed with Amicon Ultra 100 K nominal molecular weight limit devices (Millipore, Billerica, MA).

### Measurements of HIV-1 reverse transcriptase (RTase) activity

HIV-1 RTase activity was determined in triplicate samples of cell culture fluids. Supernatant (10 µl) was incubated in a reaction mixture of 0.05% Nonidet P-40, 10 µg of poly(A)/ml, 0.25 µg of oligo(dT)/ml, 5 mM dithiothreitol, 150 mM KCl, 15 mM MgCl_2_, and [^3^H]TTP in Tris-HCl buffer (pH 7.9) for 24 h at 37°C. Radiolabeled nucleotides were precipitated with cold 10% trichloroacetic acid on filter paper plates in an automatic cell harvester and washed with 95% ethanol. Radioactivity was estimated by liquid scintillation spectroscopy [18].

### RNA extraction and TaqMan real-time RT-PCR

Total RNA was isolated with TRIzol Reagent (Invitrogen) and RNeasy Mini Kit (QIAGEN Inc., Valencia, CA). Assays-on-Demand primers for human TRAIL (ID#, Hs00234356_m1), IRF-1 (ID#, Hs00971965_m1), IRF-3 (ID#, Hs00155574_m1), IRF-7 (ID#, Hs00185375_m1), and human GAPDH (ID#, 4310884E) were purchased from Applied Biosystems, Inc. (Foster City, CA). Real-time reverse-transcription polymerase chain reaction (RT-PCR) was carried out using the one-step quantitative TaqMan Real-time RT-PCR system (Applied Biosystems). TRAIL, IRF-1, IRF-3, and IRF-7 mRNA levels were determined and standardized with a GAPDH internal control, and normalized to uninfected cells using comparative ΔΔCT method. All primers used in the study were tested for amplification efficiencies and the results were similar.

### TRAIL and CCL5 ELISA

Supernatants from MDM were collected for TRAIL and CCL5 determination by ELISA (R&D Systems) as described previously [10], [43]. The sensitivity of soluble TRAIL and RANTES ELISA is 10 pg/ml. To assess concentrations of TRAIL in cell lysates of macrophages, we replaced the cell lysis buffer in the ELISA system with a lysis buffer from Pierce (Rockford, IL), which provides better lysis effect for membrane protein; the sensitivity for this assay was 100 pg/ml for TRAIL.

### Western blot analysis

Cell lysates from macrophages were prepared with M-PER Mammalian Protein Extraction Buffer (Pierce). Protein concentration was determined using the BCA Protein Assay Kit (Pierce). Protein (30 µg) was electrophoresed on pre-cast 8% SDS-PAGE and transferred to an Immuno-Blot PVDF membrane (Bio-Rad, Hercules, CA). Antibodies specific for Phospho-STAT1 at tyrosine 701, total STAT1, IRF-3, and retinoic acid-inducible gene I (RIG-I) were from Cell Signaling Technology, Inc. (Danvers, MA). Loading control β-actin proteins were detected using anti-β-actin (Sigma-Aldrich) antibodies. Membranes were treated overnight with primary antibody at 4°C followed by a horseradish peroxidase-ligand secondary anti-rabbit (Cell Signaling Technology) or anti-mouse (Cell Signaling Technology) antibody for 1 hour at room temperature. Antigen-antibody complexes were visualized by enhanced chemiluminescence (Amersham Biosciences, Piscataway, NJ) and captured with CL-X Posure™ Film (Pierce). For data quantification the films were scanned with a CanonScan 9950F scanner; the acquired images were then analyzed on a Macintosh computer using the public domain NIH image program (http://rsb.info.nih.gov/nih-image/).

### siRNA transfection

Pre-designed siRNA duplexes for IRF-1 (ID#, 115266), IRF-3 (ID#, 115222), or IRF-7 (ID#, 115481) were synthesized by Ambion Inc. (Austin, Texas). Two days post-infection MDM were transfected with 120 nM siRNA duplex for 48–96 hours in the presence of siIMPORTER (Upstate Cell Signaling Solutions, Charlottesville, VA) according to the manufacturer's instructions. A validated Silencer Negative Control #1 siRNA (Ambion Inc.) was also transfected at the same concentration as IRFs siRNA. To evaluate transfection efficiency, control and HIV-1-infected MDM were transfected with Silencer FAM-labeled Negative Control #1 siRNA (green fluorescence tagged siRNA) (Ambion Inc.). At 48 hours post-transfection, cells were incubated with Hoechst 33342 (Sigma) for nuclear staining, transfected and total cells were counted.

### Immunocytochemical assays

Human MDM were plated on 15 mm cover slips in 24-well plates. Five days after infection, cells were fixed with 4% paraformaldehyde at room temperature then incubated with methanol for 20 minutes at −20°C. Fixed cells were blocked with 3% BSA in PBS and then incubated with primary antibodies to TRAIL (human TRAIL specific polyclonal, Santa Cruz Biotechnology, Inc., Santa Cruz, CA) mixed with antibody to p24 (monoclonal mouse anti-human p24, IgG, DAKO Corp, Carpinteria, CA) at 4°C overnight. Normal mouse or rabbit IgG with matched isotype were used as negative controls for the staining. Cultures were washed and secondary antibodies, anti-mouse IgG (coupled with green dye, Alexa Flour 488, Molecular Probes, Eugene, Oregon), or anti-rabbit IgG (coupled with a info-red dye, Alexa Fluor 647, Molecular Probes) were added for 1 hour at room temperature. Nuclei DNA were labeled with Hoechst 33342 (Sigma-Aldrich) for 10 minutes at room temperature. Cover slips were mounted on glass slides with mounting medium (Sigma-Aldrich). Triple immunostaining was examined by a Bio-Rad MRC1024ES lASER scanning confocal microscope using a triple laser line and simultaneous triple display mode of the Bio-Rad LaserSharp imaging program.

### Statistical tests

Data were analyzed as means±standard deviation (SD) unless otherwise specified. The data were evaluated statistically by the analysis of variance (ANOVA), followed by the Tukey-test for paired observations. Significance was considered with a p value less than 0.05. All experiments were performed with at least three donors to account for any donor specific differences. Assays were performed at least three times in triplicate or quadruplicate.

## Results

### HIV-1-induced upregulation of TRAIL expression is predominantly membrane-associated and dependent on productive HIV-1 infection

**Figure 1:**
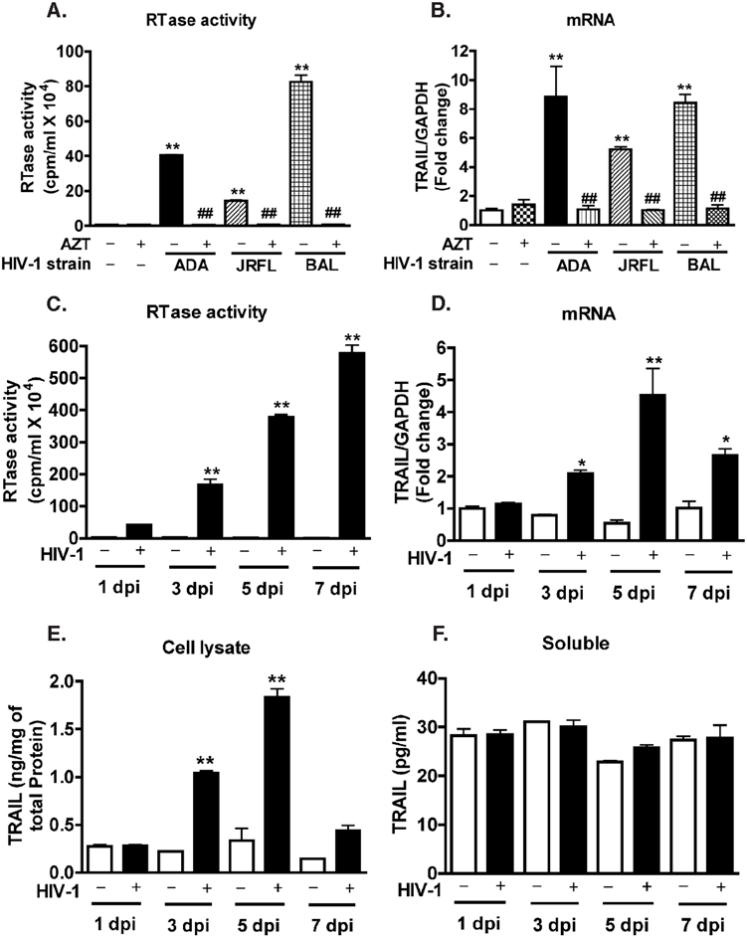
TRAIL expression in human macrophages increases when infected with HIV-1. A–B. Human MDM were infected with HIV-1_ADA_, HIV-1_JR-FL_, or HIV-1_BAL_ in the presence or absence of AZT. Cells (total RNA) and culture supernatants were collected 5 days after infection. A. Supernatants were tested for HIV-1 RTase activity. B. TRAIL expression was determined by real-time RT-PCR. Results were normalized to GAPDH expression and shown as fold change over control. ** indicates p<0.01 when compared to control; ## indicates p<0.01 when compared to corresponding HIV-1 group. C–F. Human MDM were infected with HIV-1_ADA_. Samples were collected 1, 3, 5, and 7 days after infection. C. Supernatants were tested for RTase activity. D. TRAIL expression was determined by real-time RT-PCR. E–F. TRAIL protein levels in cell lysate (E) and culture supernatants (F) were measured by TRAIL ELISA. Open bars represent control MDM and solid bars represent HIV-1-infected MDM. ** indicates p<0.01, * indicates p<0.05 when compared to the corresponding control. Data are representative of three donors.

Upregulation of TRAIL expression by HIV-1-infected macrophages has been previously reported [7], [10]; our current study investigated the mechanisms behind this upregulation. We used macrophage-tropic HIV-1 strains HIV-1_ADA_, HIV-1_JR-FL_, and HIV-1_BAL_ to infect human MDM. Five days after infection, culture supernatants were collected and HIV-1 viral infectivity was determined by the HIV-1 RTase activity assay. HIV-1_ADA_ and HIV-1_BAL_ demonstrated higher infectivity as compared to HIV-1_JR-FL_ (Fig. 1A). AZT, a HIV-1 reverse transcriptase inhibitor, completely blocked HIV-1 reverse transcription of all strains tested (Fig. 1A). The investigated macrophage-tropic viral strains all strongly upregulated TRAIL expression levels as indicated by real-time RT-PCR, and this upregulation was then blocked by reverse transcriptase inhibitor AZT (Fig. 1B). HIV-1_ADA_ strain was used thereafter and referred to as HIV-1.

To determine the effects of HIV-1 infection on TRAIL mRNA and protein levels in MDM, we used real-time RT-PCR and an ELISA-based detection system, respectively. As the infection progressed from day 1 through day 7 the HIV-1 RTase activity continued to increase (Fig. 1C). TRAIL mRNA expression was not significantly changed at 1 day post-infection but was significantly upregulated on days 3, 5, and 7 as compared to uninfected control (Fig. 1D). TRAIL mRNA upregulation peaked at day 5 and was 7.3-fold higher in HIV-infected MDM as compared to uninfected control. To determine the protein levels of TRAIL, whole-cell lysates were collected 1, 3, 5, and 7 days after HIV-1 infection and then subjected to ELISA detection. TRAIL protein levels increased starting at 3 days and peaked at 5 days after infection (Fig. 1E). After 7 days of infection, cultures underwent significant macrophage cell death (approximately 50% loss in cell viability, data not shown) and the protein level of TRAIL decreased accordingly. TRAIL ELISA was also used to measure soluble TRAIL protein within the HIV-1-infected and uninfected MDM supernatants and no significant changes in TRAIL concentrations were found (Fig. 1F). These results indicate TRAIL upregulation is associated with membrane-bound TRAIL rather than the soluble form.

**Figure 2:**
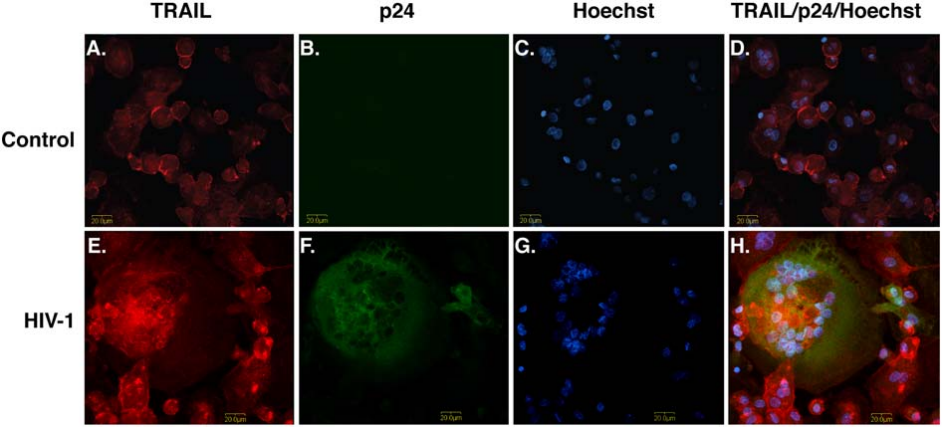
Membrane-bound TRAIL increases in HIV-1-infected macrophage culture. Human MDM were infected with HIV-1 for 5 days and then stained with antibodies to p24 (HIV-1 infection marker, green) and TRAIL (red). Nuclei (blue) were labeled with Hoechst 33342. A–D. Control uninfected MDM. E–H. HIV-1-infected MDM. Panels D and H are merged pictures of A–C and E–G, respectively. Images were acquired from a Bio-Rad MRC1024ES LASER scanning confocal microscope. Magnifications: A–H. 600×. Panels are representative of 4 separate donors.

We further specified that the membrane-bound form of TRAIL is upregulated following HIV-1 infection by using immunocytochemistry and confocal microscopy. During the progression of MDM HIV-1 infection, the percentage of infected cells continued to increase. At days 5, staining of uninfected MDM was positive for TRAIL (Fig. 2A, D) and negative for p24 (Fig. 2B, D). HIV-1-infected MDM culture showed a dramatic increase in TRAIL staining (Fig. 2E, H), particularly those MDM that were adjacent to p24-positive multinucleated giant cells expressed high levels of TRAIL surface staining (Fig. 2E, F, H). Thus, it is likely that uninfected macrophages increase TRAIL protein synthesis in response to diffusible factor(s) released by infected cells.

### HIV-1 infection induces IRF-1, IRF-7 gene expression and STAT1 phosphorylation in macrophages

**Figure 3:**
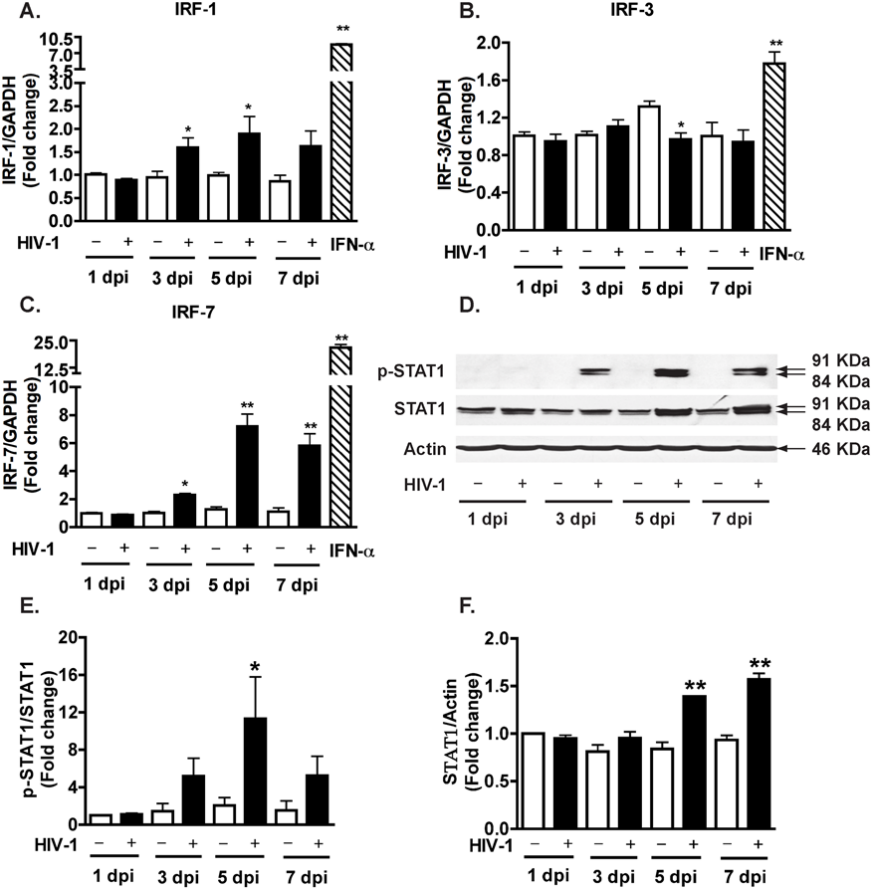
HIV-1 infection induces IRF-1 and IRF-7 gene expression and STAT1 phosphorylation at Tyr701 in macrophages. MDM were infected with HIV-1 and cell lysates and RNA were collected 1, 3, 5, and 7 days after infection. A–C. Real-time RT-PCR was used to detect IRF-1 (A), IRF-3 (B), and IRF-7 (C). Open bars represent control MDM and solid bars represent HIV-1-infected MDM. IFN-α (1000 Units/ml) was also used to stimulate MDM for 24 hours, the effect on IRFs expression is shown in each panel as the diagonal striped bar. D. Phospho-STAT1 (p-STAT1, Tyr701) and total STAT1 were detected by Western blotting. β-actin was used as a loading control. E. Levels of p-STAT1 were normalized as a ratio of p-STAT1 to STAT1 after densimetrical quantification of panel D and shown as fold change relative to control (1 dpi). F. Levels of STAT1 were normalized as a ratio of STAT1 to β-actin and shown as fold change relative to control (1 dpi). Results are shown as the average±SEM in experiments performed with three different donors. *, p<0.05 compared with day-matched control. **, p<0.01 compared to day-matched control.

To determine the molecular mechanisms that mediate TRAIL expression, we studied the signaling pathways in the macrophage innate immunity that respond to HIV-1 infection. First, we examined the regulation of the IRF transcription factors after HIV-1 infection of MDM. The expression levels of IRF-1 and IRF-7 increased upon HIV-1 infection and the increase occurred as early as 3 days after infection (Fig. 3A, C). In contrast, the expression levels of IRF-3 remained the same except at 5 days after infection, where an average 26% reduction in IRF-3 expression was found (Fig. 3B). We also treated MDM with IFN-α and observed an increase in IRF-1, IRF-3, and IRF-7 gene expression, confirming all these genes are IFN-stimulated genes (Fig. 3A–C).

IRF-1 and IRF-7 are able to induce the production of type I IFNs, which primarily activate STAT1/STAT2 signaling molecules. We next determined the phosphorylation at Tyr701 of STAT1 that is obligatory for STAT1 activation. HIV-1 induced STAT1 phosphorylation 3 days after infection, and the phosphorylation persisted for 4 additional days (Fig. 3D, E). HIV-1 infection also increased total STAT1 at 5 and 7 days after infection (Fig. 3D, F). Together, these data demonstrate that HIV-1 infection induces activation of STAT1 through phosphorylation of Tyr701 as well as an increase in total STAT1 protein levels in MDM. The similar kinetics of IRF-1, IRF-7 expression, STAT1 activation, and TRAIL production indicates these molecules may associate with the same signaling pathway.

### siRNA knockdown of IRF-1 and IRF-7 reduces TRAIL expression in HIV-1-infected macrophages

**Figure 4:**
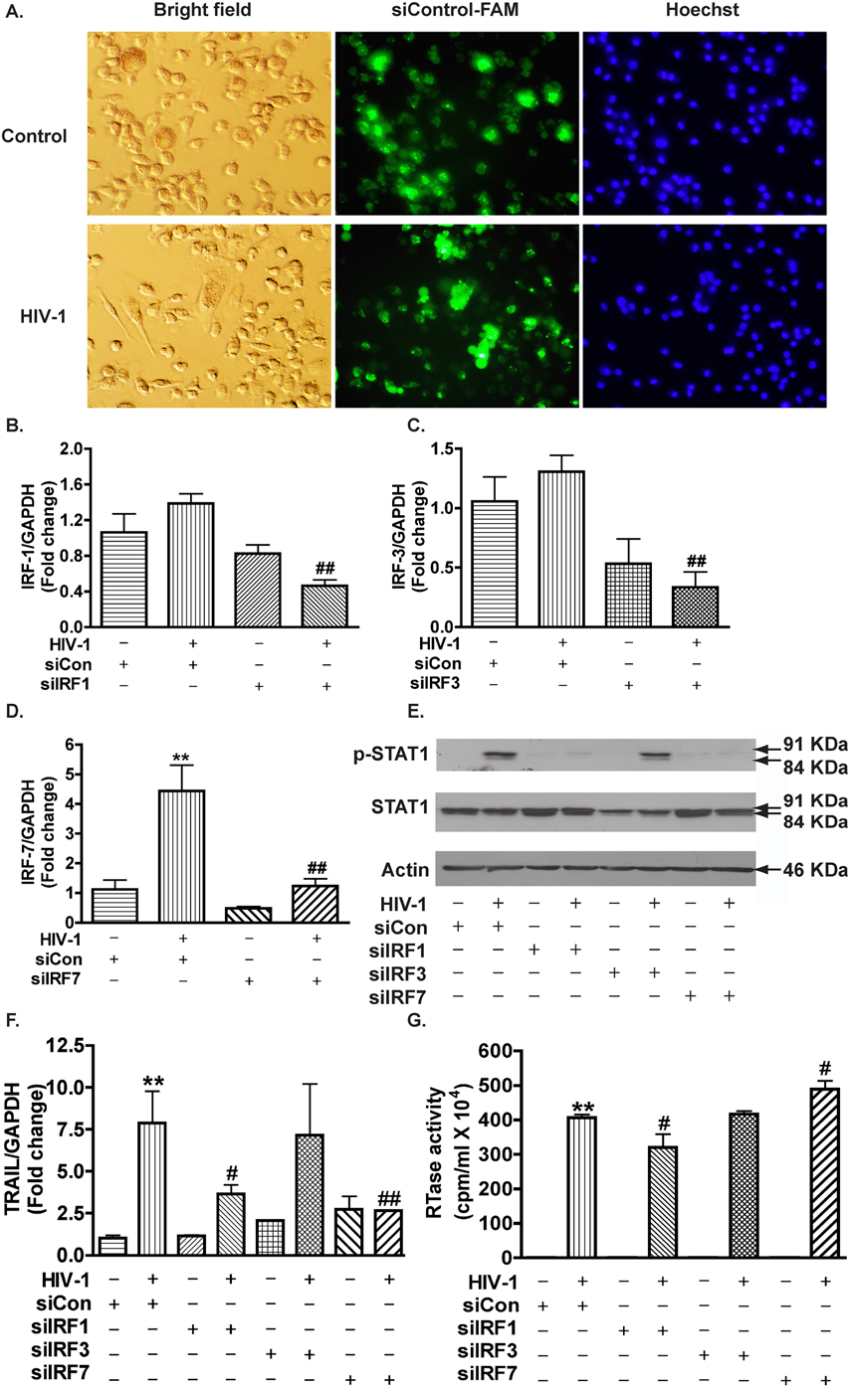
siRNA knockdown of IRF-1 and IRF-7 reduces STAT1 phosphorylation and TRAIL expression in HIV-1-infected macrophages. Two days after HIV-1 infection, MDM were transfected with siRNA for IRF-1, -3, or -7. A. Forty-eight hours later, successful transfections were confirmed by Silencer FAM-labeled Negative Control #1 siRNA transfection indicator (green). Hoechst 33258 (nucleus marker, blue) was used to visualize the total cell number. B–D. Total RNA was collected 48 hours post-transfection and mRNA levels of IRF-1(B), -3(C), or -7(D) were determined by real-time RT-PCR. E. Ninety-six hours after transfection, p-STAT1 and total STAT1 were detected by Western blotting. β-actin was used as a loading control. F. TRAIL expression levels were determined by real-time RT-PCR. Results were normalized with GAPDH and shown as the fold change over non-specific siRNA control. G. Supernatants were tested for HIV-1 RTase activity. ** indicates p<0.01 when compared to control; # indicates p<0.05 when compared to HIV group with siRNA control; ## indicates p<0.01 when compared to HIV group with siRNA control. Data are representative of three donors.

To further elucidate whether increased levels of IRFs mediate the increase in TRAIL transcription, we transfected IRF-1, IRF-3 or IRF-7 siRNA, and a nonspecific siRNA as a control in MDM cultures. siRNA was successfully delivered into both control and HIV-1-infected MDM, as demonstrated by FAM-labeled control siRNA (Fig. 4A). The transfection efficiency, measured by counting FAM-positive cells within 200 total cells, was approximately ∼70% for both control and HIV-1-infected MDM. The levels of IRF-1, IRF-3, and IRF-7 following siRNA delivery in HIV-1-infected macrophages were 33%, 14%, and 28% of non-specific siRNA-transfected HIV-infected MDM, respectively (Fig. 4B–D). STAT1 phosphorylation was significantly reduced after IRF-1 or IRF-7 knockdown in HIV-infected MDM, but was unchanged after IRF-3 knockdown when compared with control siRNA (Fig. 4E). Similarly, TRAIL expression levels remained unchanged after IRF-3 siRNA transfection, but were significantly reduced after IRF-1 siRNA transfection and were blocked by IRF-7 siRNA transfection (Fig. 4F). The viral replication in each group was monitored by the HIV-1 RTase activity assay. Knocking down IRF-1 with siRNA decreased the HIV-1 infection levels, whereas the IRF-7 knockdown increased HIV-1 infection levels. Infection levels in IRF-3 knockdown remained unchanged (Fig. 4G). These data demonstrate that IRF-1 and IRF-7 are critical to the activation of STAT1 and the upregulation of TRAIL expression in HIV-1-infected macrophages.

One unexpected finding in these IRF siRNA knockdown experiments was that IRF-3 was not required for IFN induction. It is well established that IRF3 is involved in IFN and IFN target genes inductions [31]. We further analyzed the protein knockdown of IRF-3 and found that the IRF-3 siRNA transfection reduced IRF-3 protein level by 60% in HIV-1-infected MDM (Fig. S1A). To further demonstrate the function of IRF-3 has been impaired after IRF-3 knockdown, we tested CCL5, a chemokine whose transcription is controlled by IRF-3 [44], and found that CCL5 was significantly downregulated after IRF-3 knockdown in HIV-1-infected MDM(Fig. S1B). These data suggest that knocking down IRF-3 is not sufficient to block HIV-1-induced STAT1 activation and TRAIL expression.

### IFNs potently increase macrophage TRAIL levels

**Figure 5:**
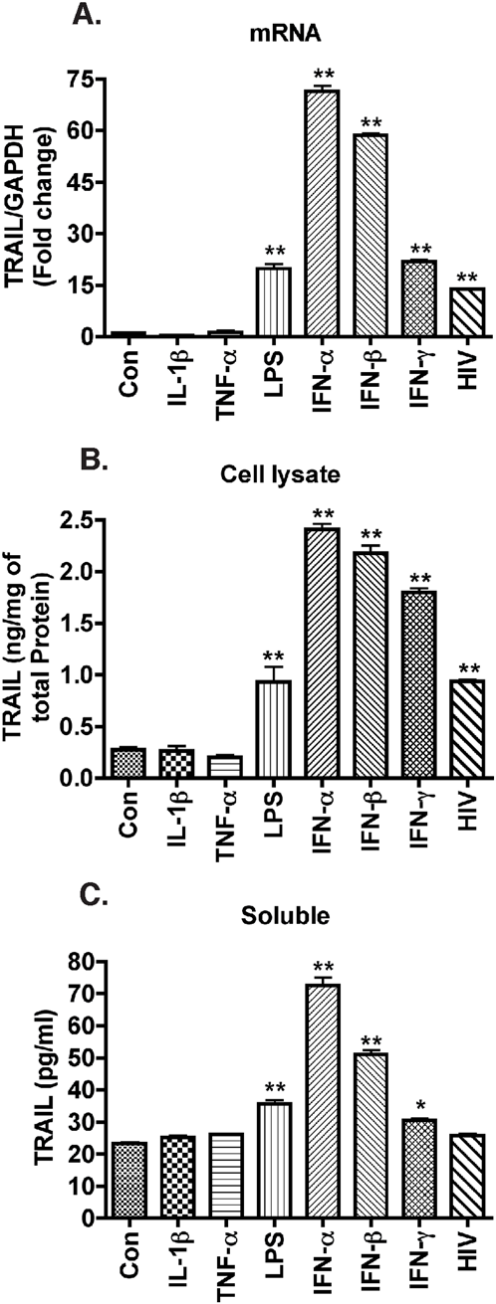
IFNs are potent stimulators of TRAIL expression in macrophages. Human MDM were infected with HIV-1 or stimulated with different inflammatory cytokines IL-1β (50 ng/ml), TNF-α (100 ng/ml), IFN-α (1000 Units/ml), IFN-β (1000 Units/ml), IFN-γ (100 ng/ml), or LPS (100 ng/ml). Cell lysates and culture supernatants were either collected 5 days following HIV-1 infection or 24 hours following stimulation. A. TRAIL expression was determined by real-time RT-PCR. B–C, TRAIL protein levels in cell lysate and culture supernatants were measured by ELISA. * indicates p<0.05, ** indicates p<0.01 when compared to control. Data are representative of three donors.

Macrophages release various inflammatory cytokines such as IL-1β and TNF-α upon HIV-1 infection [45], [46]. We previously reported LPS- and IFN-γ-treated macrophages have higher levels of cell-surface TRAIL through analysis by flow cytometry [10]. In this study, we used quantitative TRAIL ELISA and evaluated whether TRAIL synthesis by macrophages is mediated by inflammatory cytokines, such as IL-1β and TNF-α. We treated MDM with a panel of inflammatory cytokines and LPS. LPS has been reported to increase both forms of TRAIL in MDM, and it served as positive control in our TRAIL detection system [47]. TRAIL mRNA increased in response to LPS stimulation (Fig. 5A), and TRAIL protein increased modestly in the cell lysates and supernatants in response to LPS treatment (Fig. 5B, C). Treatment of MDM with individual cytokines IL-1β (50 ng/ml) or TNF-α (100 ng/ml) did not change membrane-bound or soluble TRAIL protein levels nor were TRAIL mRNA levels affected. In contrast, IFNs, including IFN-α, IFN-β, and IFN-γ, caused a significant increase in TRAIL protein and mRNA levels (Fig. 5A–C). Macrophages do not typically produce type II IFNs, thus type I IFNs remained the focus of our investigation.

### Increased TRAIL expression in HIV-1-infected macrophages is dependent on type I IFN activity

**Figure 6:**
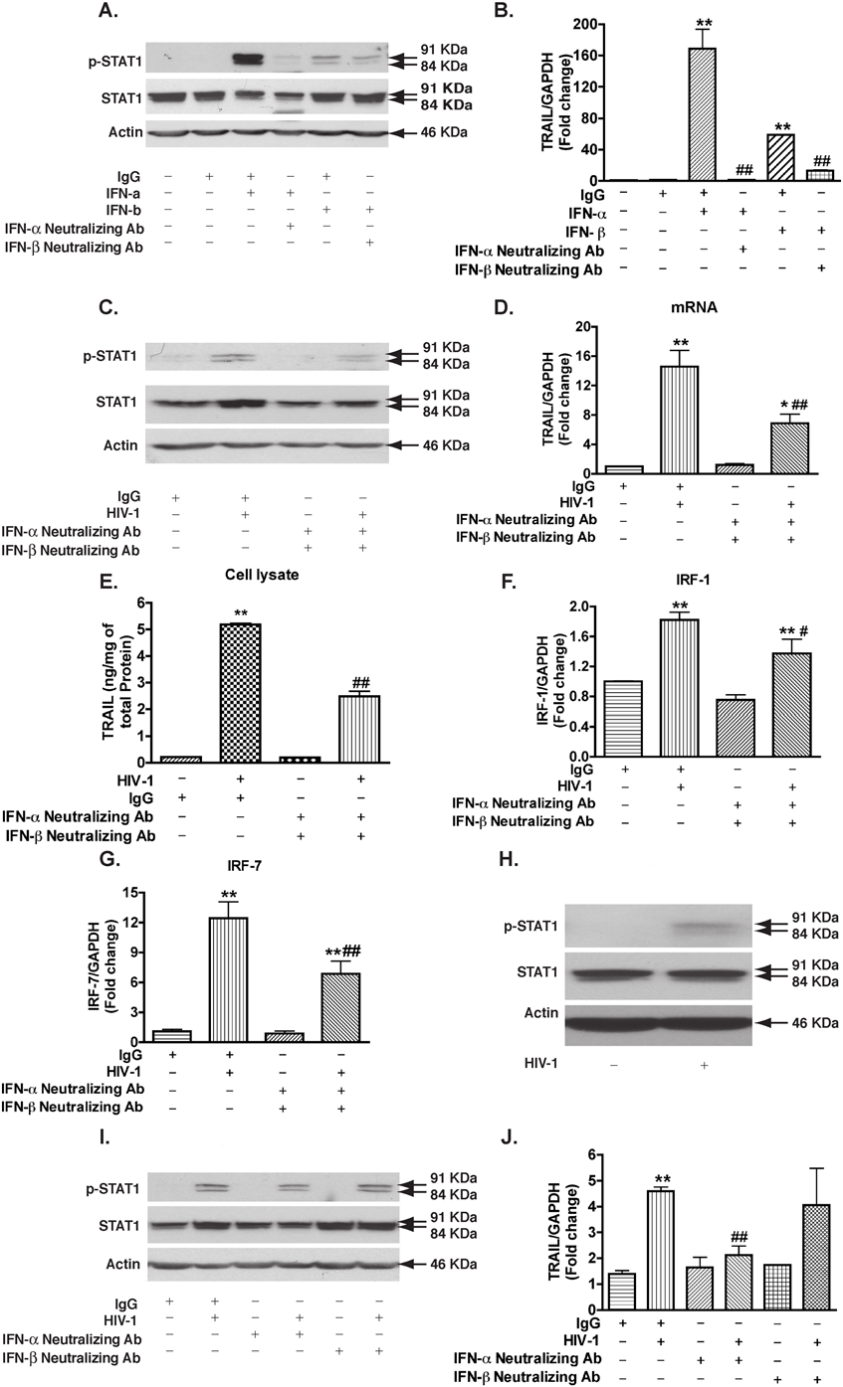
Type I interferon from HIV-1-infected macrophages induces STAT1 phosphorylation, IRF-1, IRF-7, and TRAIL expression. A. MDM were treated with IFN-α (1000 Units/ml) or IFN-β (1000 Units/ml) with or without their corresponding neutralizing antibodies. Cell lysates were collected 2 hours later and subjected to Western blotting for p-STAT1 and STAT1. β-actin was used as a loading control. B. 24 hours after the treatment, TRAIL expression was determined by real-time RT-PCR. ** denotes p<0.01 compared with IgG control; ## indicates p<0.01 compared with the corresponding IFN group. Experiments are representative of duplicate assays from two different donors. C–G. Human MDM were infected with HIV-1 for 5 days with or without type I IFNs-neutralizing antibodies and total RNA and cell lysates were collected. C. Cell lysates were subjected to Western blotting for p-STAT1 and STAT1. D. TRAIL expression was determined by real-time RT-PCR. E. TRAIL protein levels in cell lysates were detected by ELISA. Experiments are representative of three different donors. *, p<0.05 compared with neutralizing antibodies treatment. F–G. IRF-1(F) and IRF-7(G) expression were determined by real-time RT-PCR. Results were shown as the average±SEM in experiments performed with three different donors. H. Supernatants from HIV-1 culture were collected 5 days after infection and filtered with 100 k centrifugal filter device. The flow-through was transferred to control MDM for 2 hours and STAT1 phosphorylation was determined by Western blotting. I. Supernatants from HIV-1 culture were transferred to control MDM culture with or without IFN-α- or IFN-β- neutralizing antibodies. Cell lysates were collected 2 hours later and subjected to Western blotting for p-STAT1 and STAT1. J. Twenty-four hours after the treatment, TRAIL expression was determined by real-time RT-PCR. ** indicates p<0.01 when compared to IgG control; #, p<0.05, ##, p<0.01 compared with IgG/HIV-1 group.

STAT1 activation is essential for the cells to response to type I IFNs [48], [49]. STAT1 activation in MDM by HIV-1-infection suggests there are type I IFNs in the culture supernatant acting in autocrine and paracrine manner. To test whether type I IFNs were responsible for STAT1 activation and the subsequent increase in TRAIL levels after HIV-1-infection of MDM, type I IFN-neutralizing antibodies were administered. The type I IFN-neutralizing antibodies worked effectively because inhibition of IFN-α-induced STAT1 phosphorylation was found to be 92% for IFN-α-neutralizing antibodies, and inhibition of IFN-β-induced STAT1 phosphorylation (54%) were observed by IFN-β-neutralizing antibodies (Fig. 6A). In addition, TRAIL expression was reduced by 97% and 79% after the treatment with the IFN-α- or IFN-β-neutralizing antibodies, respectively (Fig. 6B). We then added type I IFN-neutralizing antibodies to HIV-1-infected MDM every 24 hours after HIV-1 infection until the fifth day. The neutralizing antibodies did not appear to change the RTase activity, but partially blocked HIV-1-stimulated STAT1 phosphorylation (54% inhibition, Fig. 6C). Moreover, membrane-bound TRAIL and TRAIL levels were reduced by the neutralizing antibodies as compared to IgG control antibody (Fig. 6D, E). The addition of neutralizing antibodies against type I IFNs partially blocked HIV-1-induced IRF-1 and IRF-7 expression (Fig. 6F, G). These data further support the hypothesis that increased IRF-1, IRF-7, and TRAIL expression after HIV-1 infection is reliant on type I IFNs.

To validate type I IFNs were the diffusible factors that regulated TRAIL, we transferred supernatant from HIV-1-infected MDM to uninfected MDM. Exposure of control MDM to HIV-1-infected supernatants for 2 hours led to a dramatic activation of STAT1 similar to that seen in HIV-1-infected MDM (Figs. 3 and 6I). Because binding of HIV-1 virions or gp120 may also activate STAT1, we used centrifugal filters with 100 kDa molecular weight pores to separate HIV-1 virions and gp120 from lower molecular weight compounds. After filtration, HIV-1 RTase activity was completely lost (data not shown), suggesting successful removal of virions. The lower molecular weight fraction after filtration induced strong STAT1 phosphorylation (Fig. 6H), confirming diffusible factors (<100 kDa) are capable of activating STAT1. Interestingly, IFN-α-neutralizing antibody reduced STAT1 activation (48% inhibition) and significantly reduced TRAIL expression levels, whereas IFN-β-neutralizing antibody treatment did not cause a statistically significant decrease in STAT1 activation or TRAIL expression (Fig. 6I, J). These data demonstrate that type I IFNs, likely IFN-α, is released by macrophages upon HIV-1 infection resulting in increased TRAIL levels.

### STAT1 is essential for IRFs and TRAIL expression in HIV-1-infected macrophages

**Figure 7:**
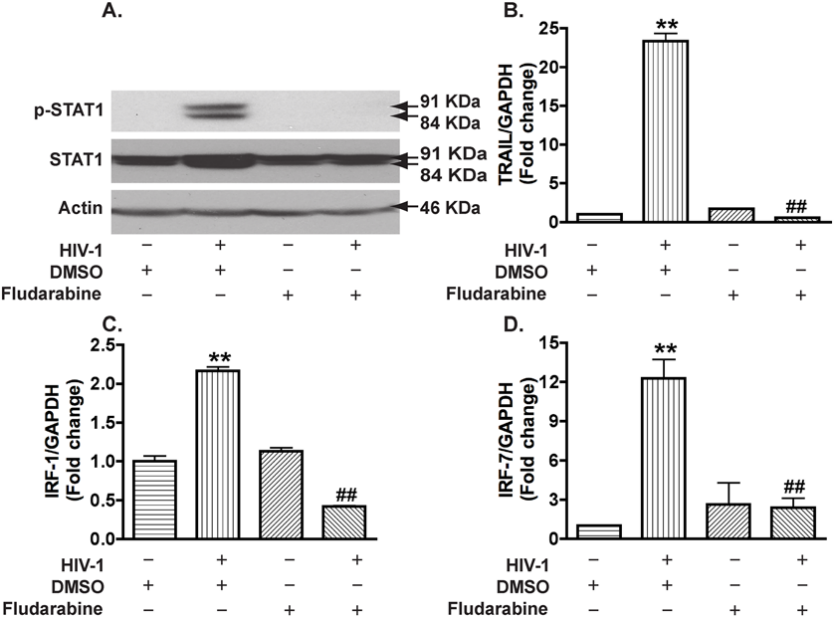
Fludarabine blocks HIV-1-induced STAT1 activation and gene expression of IRF-1, IRF-7, and TRAIL in macrophages. A. MDM were treated with fludarabine at 1 µM 3 days after infection. P-STAT1 and total STAT1 were detected by Western blotting at 5 days after infection. β-actin was used as a loading control. B–D. Real-time RT-PCR was used to detect TRAIL (B), IRF-1 (C), and IRF-7 (D). **, p<0.05 compared with DMSO control. ##; p<0.01 compared to DMSO-treated HIV-1 group.

We next determined whether STAT1 modulates HIV-1-induced TRAIL expression in MDM. Fludarabine, a compound that has been shown to specifically inhibit STAT1 activation and induce loss of STAT1 mRNA and proteins [50], was used to block HIV-1-mediated STAT1 activation. When used at 1 µM, fludarabine abolished HIV-1-induced phosphorylation of STAT1 as well as HIV-1-induced increase in total STAT1 (Fig. 7A). Accordingly, HIV-1-induced gene expression of TRAIL (Fig. 7B), IRF-1 (Fig. 7C), and IRF-7 (Fig. 7D) were completely blocked by fludarabine treatment. These results suggest that STAT1 is essential for IRF-1, IRF-7, and TRAIL expression in HIV-1-infected macrophages.

## Discussion

**Figure 8:**
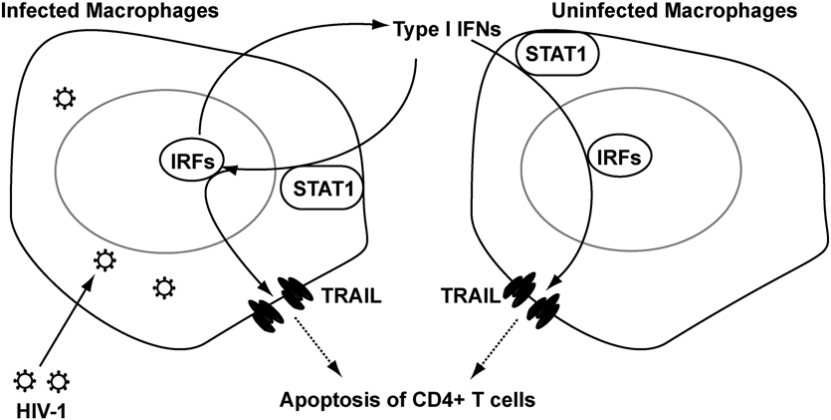
A feedback loop among Type I IFNs and IRFs regulates TRAIL Expression in HIV-1-infected macrophages. A positive feedback loop exists among intracellular IRFs gene expression and soluble type I IFNs induction in macrophages during HIV-1 infection. HIV-1 infection induces IRF-1 and IRF-7 gene expression. IRF-1 and IRF-7 promotes type I IFNs activities and activate STAT1. Type I IFNs diffuse and further promote STAT1 activation and IRF-1 and IRF-7 expression in uninfected macrophages. Blocking of either IRF-1, IRF-7, type I IFNs, or STAT1 reduces TRAIL expression. TRAIL has been reported to mediate the apoptosis of CD4+ T cells and participate in HIV-1 pathogenesis.

The molecular mechanisms of TRAIL induction by HIV-1 in macrophages are not completely understood. Here we investigated the regulation of TRAIL as well as the upstream molecular events responsible for TRAIL induction in HIV-1-infected macrophages. We demonstrated that upregulation of TRAIL expression in HIV-1-infected MDM was predominantly membrane-associated (Fig. 1, 2). HIV-1 infection induced IRF-1, IRF-7 gene expression and activated STAT1 in macrophages (Fig. 3). IRF-1 and IRF-7 promoted Type I IFNs production and subsequent STAT1 activation (Fig. 4). Type I IFNs and STAT1 activation further increased IRF-1 and IRF-7 gene expression (Fig. 6, 7). Blocking signaling factors, including IRF-1, IRF-7, type I IFNs, or STAT1, significantly reduced TRAIL gene expression (Fig. 4, 6, 7). These data provide insight to the detailed regulation of TRAIL and identify IRF-1, IRF-7, type I IFNs, and STAT1 as critical signaling intermediates for TRAIL induction. Although IRFs have been reported to regulate type I IFNs, we were surprised to find that IRF-3 is not as critical as IRF-1 or IRF-7 in the signaling cascade (Fig. 4). Instead, a positive feedback loop between intracellular IRF1, IRF-7, STAT1 and soluble type I IFNs exists and cooperatively regulates TRAIL in HIV-1-infected macrophages (see scheme in Fig. 8).

We [10], [11], as well as others [6]–[9] have identified TRAIL-induced apoptosis in several cell types during HIV-1 infection. The exact pathological consequences of the increased membrane-bound TRAIL in macrophages and in other cell types remain to be established. Given that TRAIL preferentially kills HIV-1-infected macrophages, it is plausible that the initial increase in TRAIL is part of the innate immune response directed toward the elimination of HIV-1-infected cells. Other unexpected target of TRAIL, particularly uninfected CD4+ T cells, may add to the complexity of TRAIL-mediated cell death. The increased membrane-bound form of TRAIL in macrophages may team together with either membrane-bound or soluble form of TRAIL in monocytes and CD4+ T cells, possibly causing apoptosis of bystander CD4+ T cells. This adverse effect of TRAIL on the adaptive immune system during HIV-1 infection may help to explain why HIV-1 persists even in the presence of elevated soluble and membrane-bound TRAIL.

Members of the IRF family are important antiviral transcription factors. IRF-3 and IRF-7 participate in immune responses and are primarily associated with Type I IFNs [30]–[32]. In addition, IRF-1, IRF-5, and IRF-8 can also contribute to type I IFNs induction (for review, see [51]). Increased IRF-1 expression has been reported in HIV-1-infected Jurkat and primary CD4+ T cells [52]. IRF-7 is increased in HIV-1-infected individuals in plasmacytoid dendritic cells, another mononuclear phagocyte cell type and the major IFN-producing cells [36]. However, limited information on the regulation and function of IRFs in HIV-1-infected macrophages has been reported to date. Our data show increased IRF-1 and IRF-7 expression in HIV-1-infected macrophages (Fig. 3), and that IRF-7 knockdown in macrophages facilitated HIV-1 replication (Fig. 4F), highlighting the importance of IRF-7 in the antiviral response of macrophages. In contrast, knockdown of IRF-1 inhibited HIV-1 replication (Fig. 4F). The difference between IRF-1 and IRF-7 on HIV-1 replication may be due to the requirement of IRF-1, but not IRF-7, for full NF-κB transcriptional activity at the HIV-1 long term repeat enhancer [53]. The unique roles of IRF-1 in the enhancement of HIV-1 replication and induction of death ligand TRAIL provide a potentially novel therapeutic target, and inhibition of IRF-1 may simultaneously reduce HIV-1 viral load and alleviate macrophage-mediated HIV-1 pathogenesis.

IRF-1 and IRF-3 have been shown to regulate TRAIL transcription in tumor cell lines [38]–[40]. More recently, overexpression of IRF-7 has been found to enhance TRAIL transcription in macrophages [41]. When we applied siRNA to knockdown IRF-1, IRF-3, or IRF-7 gene expression in human macrophages, the increase of TRAIL expression by HIV-1 infection was reduced by the IRF-1 and the IRF-7 knockdown, but not by the IRF-3 knockdown (Fig. 4G). This is, to the best of our knowledge, the first report of IRFs knockdown in HIV-1-infected macrophages. Knockdown of IRF-1 and IRF-7 reduced STAT1 phosphorylation, an essential component for type I IFNs responsiveness (Fig. 4E). However, type I IFNs-neutralizing antibodies did not completely block TRAIL upregulation in HIV-1-infected culture (Fig. 6D, E), suggesting the involvement of a type I IFNs-independent pathway in the induction of TRAIL. These type I IFNs-dependent and -independent mechanisms may work concomitantly in HIV-1-infected culture to induce TRAIL expression. Our analysis also found that IRF-5 gene expression could be induced upon HIV-1 infection in macrophages but in lower abundance. In addition, IRF-8 gene expression was not induced by HIV-1 infection but was expressed at a higher amount. The role of these additional members of IRFs in the type I IFNs production and TRAIL regulation remains to be elucidated.

The upstream molecular mechanisms resulting in the activation of IRF-1 and IRF-7 during viral infection have begun to be elucidated in recent years. Toll-like receptors (TLRs) and RIG-I-like receptors are two separate classes of pattern-recognition receptors (PRRs) that detect viral infection and initiate signaling cascades including IRFs and type I IFNs (for review, see [54]). However, TLR signaling cascade, which can be activated in plasmacytoid dendritic cells, fails to promote activation in macrophages in response to HIV-1 [55]. We suspect that other viral sensing pathways may lead to IRFs activation in HIV-1-infected macrophages. We tested RIG-I, a TLR-independent PRRs, and found that HIV-1 infection increased RIG-I protein levels, and the increase occurred as early as one day after infection (Fig. S2). Activation of RIG-I leads to a signaling results in the activation of IRF-3 and IRF-7 [56]–[58]. Furthermore, melanoma differentiation-associated gene 5, another RIG-I like receptor, has been reported to activate IRF-1, -3, and -7 [57], [59]. Despite our extensive studies, we cannot exclude the potential roles of TLRs in the regulation of IRF-1 and IRF-7 during HIV-1 infection. The upregulation of RIG-I by HIV-1 infection in macrophages is novel and interesting, however its relationship with IRF-1 and IRF-7 regulation remains the subject of further investigation.

Recently HIV-1 accessory proteins, VPR and Vif, have been reported to degrade IRF-3 through ubiquitin-associated proteosome pathway [60]. If IRF-3 degradation occurred in our HIV-1-infected MDM culture, it could skew the interpretations of our current results. To discount this, we have tested the IRF-3 protein levels by Western blotting and found no dramatic degradation of IRF-3 in our MDM during the HIV-1 infection course (data not shown). In fact, there is a transient increase of IRF-3 at 5 days post HIV-1 infection (Fig. S1A). This is more comparable with a previous publication, which showed neither degradation nor activation of IRF-3 in HIV-1-infected macrophages [55]. This inconsistency in the literature may be explained by the differences in cellular models and stages of HIV-1 infection. In addition, a cell type-specific role for IRF-1 to supplant the requirement for IRF-3 in macrophages has been reported recently [61]. Whether upregulation of IRF-1 and IRF-7 could potentially restore the function of IRF-3 awaits future investigation.

Our results identify type I IFNs as a critical component of the signaling cascade regulating TRAIL expression. In HIV-1 infection, type I IFNs are produced mainly by plasmacytoid dendritic cells and in lower amounts by monocytes and macrophages [23], [24], [62]. Interestingly, type I IFNs have been tested in clinical trials for HIV-1 treatment and resulted in a transiently decreased viral load and increased hematologic toxicity and peripheral neuropathy [63]. Although type I IFNs activate macrophages and improve the immune function of macrophages, our data found endogenous type I IFNs do not significantly decrease viral replication in infected MDM cultures (data not shown). This contradiction suggests that there is a complex interaction between HIV-1 and macrophages, where the innate immune response may contribute to viral replication.

STAT1 activation in HIV-1-infected MDM and peripheral blood mononuclear cells has been reported and correlated with HIV pathogenesis [64], [65]. STAT1 activation seems to primarily be involved in the response to type I and II IFNs and involves phosphorylation of Tyr 701 and/or Ser727 [48], [49]. Tyr701 is obligatory for STAT1 activation, while Ser727 may be required for the maximal induction of STAT1-mediated gene activation [26], [66]. We have demonstrated that HIV-1 infection increased STAT1 phosphorylation at Tyr701 and total STAT1 expression (Fig. 3) and that the activation of STAT1 is essential for IRF-1 and IRF-7 expression and TRAIL induction (Fig. 7). The potential mechanism(s) linking upstream STAT1 activation to IRF-1 and IRF-7 is not investigated in the current study. The activation of STAT1 could directly bind to the IRF-1 or IRF-7 promoter and turn on gene transcription [67]–[69]. Moreover, a reciprocal build up between IRF and IFN in the later stages of infection may contribute to the changes of gene expression and STAT1 activation. In addition, it should be noted that other cytokines such as epidermal growth factor, platelet-derived growth factor, and interleukin-6, along with HIV-1 virions and viral proteins such as gp120, Tat, and Nef may be secreted by HIV-1-infected macrophages thereby mediating type I IFNs production or STAT1 activation [7], [8], [65], [70]–[72]. Nevertheless, our data strongly support a critical role for IRF-1, IRF-7, and type I IFNs in the induction of macrophage STAT1 activation during HIV-1 infection.

Biologically active forms of TRAIL include membrane-bound TRAIL and soluble TRAIL [4], [28], [73]. In our study, both transcription and membrane-bound levels of TRAIL were significantly increased in HIV-1-infected or IFN-treated MDM but not by inflammatory cytokines TNF-α- or IL-1β-mediated activation (Fig. 5). Notably, soluble TRAIL was secreted by macrophages following type I interferon treatment but not by HIV-1-infected macrophages (Fig. 1). This is consistent with a previous report that there was no soluble TRAIL production upon exposure to HIV-1 [5]. The low production of soluble TRAIL despite the dramatic increase of transcription and membrane-bound TRAIL is probably cell type-specific and the regulation mechanisms warrants future investigation.

In summary, our current study revealed the signaling mechanisms of TRAIL upregulation in HIV-1-infected macrophages. The role of IRF-1, IRF-7, type I IFNs, and STAT1 in the regulation of TRAIL during HIV-1 infection of macrophages is important and adds to our understanding of pathogenesis of HIV-1. Identifying cytotoxicity in the antiviral response to type I IFNs and its signaling mechanism would potentially provide targets for therapeutic interventions for HIV-1 infection.

## Supporting Information

Figure S1 siRNA knockdown of IRF-3 reduces CCL5 production in HIV-1-infected macrophages. Two days after HIV-1 infection, MDM were transfected with siRNA for IRF-3. A. Ninety-six hours after transfection, IRF-3 was detected by Western blotting. β-actin was used as a loading control. Levels of IRF-3 were normalized as a ratio of IRF-3 to β-actin after densimetrical quantification and shown as fold change relative to non-specific siRNA control. B. CCL5 levels were determined by ELISA. ** indicates p<0.01 when compared to non-specific siRNA control; ## indicates p<0.01 when compared to HIV group with siRNA control. Data are representative of three donors.
(0.85 MB TIF)

Figure S2 Infection with HIV-1 induces an increase of RIG-I in macrophages. MDM were infected with HIV-1 and cell lysates were collected 1, 3, 5, and 7 days after infection. RIG-I was detected by Western blotting and β-actin was used as a loading control. Levels of RIG-I were normalized as a ratio of RIG-I to β-actin after densimetrical quantification and shown as fold change relative to control (1 dpi). Results are shown as the average±SEM in experiments performed with five different donors. *, p<0.05 compared with day-matched control. **, p<0.01 compared to day-matched control.
(0.72 MB TIF)
