# Supplementary figures and images for: Type I Interferons and Interferon Regulatory Factors Regulate TNF-Related Apoptosis-Inducing Ligand (TRAIL) in HIV-1-Infected Macrophages
Source: PLoS One. 2009 Apr 30;4(4):e5397. doi: 10.1371/journal.pone.0005397 (PMC2672636; doi:10.1371/journal.pone.0005397)

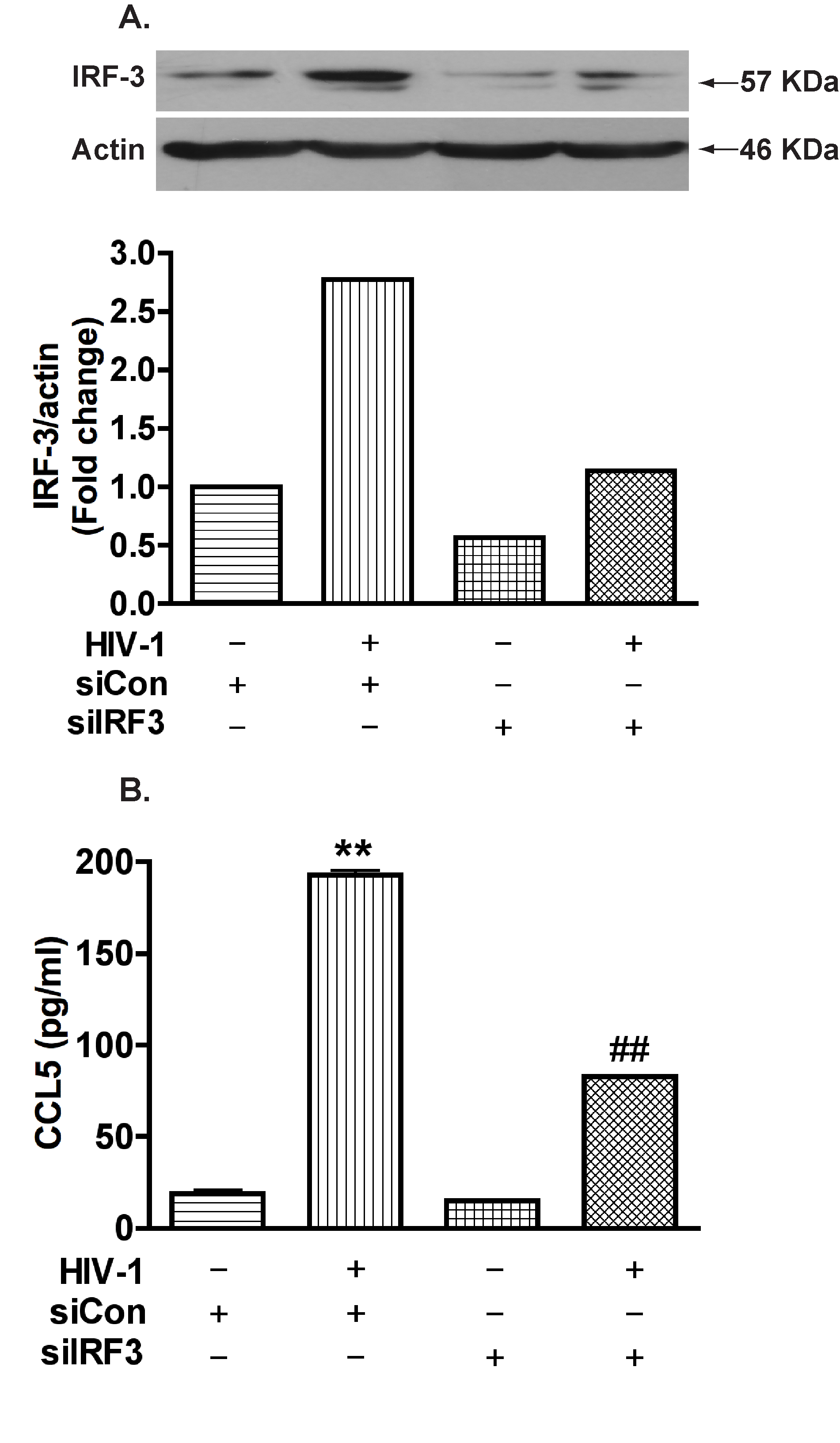

Supplement: Figure S1 — siRNA knockdown of IRF-3 reduces CCL5 production in HIV-1-infected macrophages. Two days after HIV-1 infection, MDM were transfected with siRNA for IRF-3. A. Ninety-six hours after transfection, IRF-3 was detected by Western blotting. β-actin was used as a loading control. Levels of IRF-3 were normalized as a ratio of IRF-3 to β-actin after densimetrical quantification and shown as fold change relative to non-specific siRNA control. B. CCL5 levels were determined by ELISA. ** indicates p<0.01 when compared to non-specific siRNA control; ## indicates p<0.01 when compared to HIV group with siRNA control. Data are representative of three donors. (0.85 MB TIF) [file pone.0005397.s001.tif]

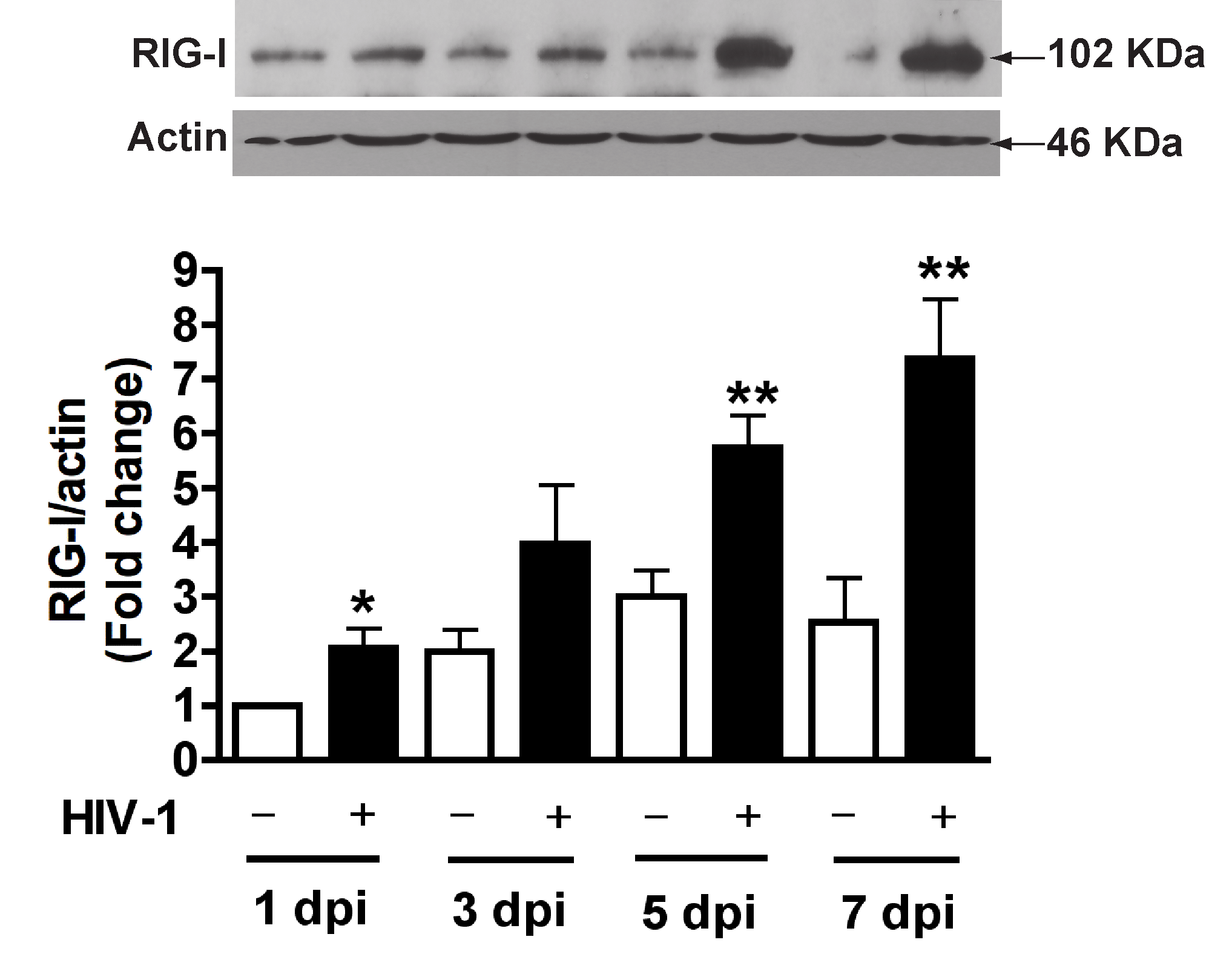

Supplement: Figure S2 — Infection with HIV-1 induces an increase of RIG-I in macrophages. MDM were infected with HIV-1 and cell lysates were collected 1, 3, 5, and 7 days after infection. RIG-I was detected by Western blotting and β-actin was used as a loading control. Levels of RIG-I were normalized as a ratio of RIG-I to β-actin after densimetrical quantification and shown as fold change relative to control (1 dpi). Results are shown as the average±SEM in experiments performed with five different donors. *, p<0.05 compared with day-matched control. **, p<0.01 compared to day-matched control. (0.72 MB TIF) [file pone.0005397.s002.tif]
